# The Effect of Binaural Beats on Visuospatial Working Memory and Cortical Connectivity

**DOI:** 10.1371/journal.pone.0166630

**Published:** 2016-11-28

**Authors:** Christine Beauchene, Nicole Abaid, Rosalyn Moran, Rachel A. Diana, Alexander Leonessa

**Affiliations:** 1 Center for Dynamic Systems Modeling and Control, Department of Mechanical Engineering, Virginia Polytechnic Institute and State University, Blacksburg, Virginia, United States of America; 2 Department of Biomedical Engineering and Mechanics, Virginia Polytechnic Institute and State University, Blacksburg, Virginia, United States of America; 3 Department of Engineering Mathematics, University of Bristol, Clifton, Bristol, United Kingdom; 4 Department of Psychology, Virginia Polytechnic Institute and State University, Blacksburg, Virginia, United States of America; Universidad de Salamanca, SPAIN

## Abstract

Binaural beats utilize a phenomenon that occurs within the cortex when two different frequencies are presented separately to each ear. This procedure produces a third phantom binaural beat, whose frequency is equal to the difference of the two presented tones and which can be manipulated for non-invasive brain stimulation. The effects of binaural beats on working memory, the system in control of temporary retention and online organization of thoughts for successful goal directed behavior, have not been well studied. Furthermore, no studies have evaluated the effects of binaural beats on brain connectivity during working memory tasks. In this study, we determined the effects of different acoustic stimulation conditions on participant response accuracy and cortical network topology, as measured by EEG recordings, during a visuospatial working memory task. Three acoustic stimulation control conditions and three binaural beat stimulation conditions were used: None, Pure Tone, Classical Music, 5Hz binaural beats, 10Hz binaural beats, and 15Hz binaural beats. We found that listening to 15Hz binaural beats during a visuospatial working memory task not only increased the response accuracy, but also modified the strengths of the cortical networks during the task. The three auditory control conditions and the 5Hz and 10Hz binaural beats all decreased accuracy. Based on graphical network analyses, the cortical activity during 15Hz binaural beats produced networks characteristic of high information transfer with consistent connection strengths throughout the visuospatial working memory task.

## Introduction

Findings from the cognitive neuroimaging literature show that the integration of regional neuronal activity, in the form of coordinated network processing, is required for complex cognition (e.g. memory tasks involve prefrontal, temporal, and sensory processes). In memory tasks, for example, these interactions across regions are thought to be reflected in the coupling across multiple EEG oscillatory bands, particularly at theta (4Hz-8Hz) and gamma (25Hz-40Hz) frequencies [1–4]. Networks describing the interconnections between these cortical regions exhibit a high degree of randomness and modularity, but relatively low heterogeneity. The network properties, which are conserved over all scales, include small world degree distributions, short path lengths, modularity, hierarchy, hub nodes, and robustness [5]. While the native networks associated with memory have been explored, their response to noninvasive stimulation remains to be fully characterized. The study presented here examines the network properties of brain regions involved in working memory with the goal of understanding how environmental stimuli, in this case acoustic stimulation with specific oscillatory synchrony, can modulate cognitive processing.

Working memory is the system in control of online processing and organization of information for successful reasoning, comprehension, and goal directed behavior [6, 7]. Individuals exhibit a capacity limit on the number of items that can be simultaneously retained in working memory. Neuroanatomically, the network governing working memory is distributed over a large part of the brain. In particular, working memory tasks involving visuospatial information activate areas of the prefrontal cortex (PFC) and are often right lateralized [8, 9]. In addition, throughout working memory maintenance, the prefrontal and parietal neuronal ensembles are activated simultaneously [10–12]. Increased difficulty on a working memory task is associated with increased connectivity between prefrontal and parietal areas [13].

Cortical activity can be noninvasively recorded from the scalp using EEG. Since EEG is noisy, non-linear, and non-stationary, phase synchronization is well suited for cortical network determination. Phase synchronization is a statistical method to measure the interdependence of two oscillators and it has been applied in the fields of nonlinear dynamics and chaotic systems [14–16]. Short-range, or local, phase synchronization within the brain can be interpreted as creating regional “perceptual binding” [17]. Long-range phase synchronization, between regions, is thought to sub-serve motor planning [18, 19], emotion [20, 21], and memory [22–25].

Previous research has shown that an increase in electrocortical phase synchronization across the cortex facilitates neural communication, promotes neural plasticity, and supports working memory [25]. Synchronized firing of presynaptic neurons increases the firing rate of the postsynaptic neuron because multiple simultaneous inputs sum to increase the likelihood that the postsynaptic neuron’s threshold will be reached [26]. Synchronous gamma oscillations are confined to local neuronal areas, whereas theta synchronization is effective across long distances (i.e. disparate regions of the brain) [27–29]. Successful encoding of information during a memory task requires increased phase synchronization [22, 30–36]. The phase synchronization, within the theta band, between prefrontal and parietal regions during a working memory task is sustained during encoding, maintenance, and retrieval and this synchrony increases with memory load [28, 37]. Stimulation-induced beta and gamma synchronization produces increased coherence between frontal and parietal areas during working memory maintenance [38, 39].

Synchronization can be induced noninvasively via presentation of specific auditory stimuli, called binaural beats (BB). Binaural beats requires the presentation of two different tones to the ears [40]. This procedure causes a third phantom binaural beat, whose frequency is equal to the difference of the two presented tones, to be produced within the Inferior Colliculus (IC) located in the auditory pathway [41–44]. The overall phase difference is preserved from the IC to the auditory cortex by periodic neural firing at the binaural beat frequency [45]. The highest amount of synchronization in the auditory cortex due to binaural beats occurs within the beta band at 16Hz [42].

Previous work has demonstrated that binaural beats can affect cortical responses across frequency bands. Within the gamma band, the largest EEG steady state responses occurred with a binaural beat of 40Hz and primarily activated the frontal and parietal lobes [46–49]. In addition, binaural beat stimulation in the beta band at 18.5Hz increased EEG magnitude by 21% [50]. Areas of the cortex entrained by theta band binaural beats include parietal, frontal, and temporal areas [51–54]. However, binaural beats can influence activity outside their respective frequency band and this effect is not well characterized. For example, Gao et al. reported that, during either delta or alpha binaural beat stimulation, the EEG power increased in their respective band. However, in addition to the stimulated band, the relative EEG power increased in the theta and alpha bands as well [55].

A previous study, by Ioannou et al., investigated the impact of binaural beats on phase synchrony measures in both musicians and non-musicians. They found that binaural beat stimulation in the alpha band created the highest steady state responses in both groups. In addition, they determined that listening to low frequency binaural beats had a significant impact on the structure of the cortical connectivity network in the alpha band [56]. This work suggests that binaural beats will be able to significantly impact the network topology for improving memory.

Although binaural beats offer a noninvasive and easily administered stimulus, their effect on working memory has been explored in only a small number of studies. Kennerly investigated the effect of binaural beats on performance during memory span tasks [57]. The author concluded that the binaural beat groups performed significantly better when compared to the control group. Fernandez et al. tested the effects of binaural beats on verbal working memory [58]. Participants performed significantly better on a word recall task when listening to 5Hz binaural beat when compared to 13Hz. Lane et al. tested participant performance during a 1-back working memory test while listening to either theta or beta range binaural beats [59]. While listening to binaural beats in the beta frequency range, participants showed improvement in target detection, and decreased false alarms, task-related confusion, and fatigue. Although previous studies suggest that binaural beats offer noninvasive manipulation of brain activity that produces behavioral changes in working memory performance, prior studies have not investigated the neural mechanisms that drive the behavioral changes.

The goal of this study is to determine the effect of binaural beats on cortical connectivity and associate any changes in cortical network properties with behavioral performance during a visuospatial working memory task. In particular, we will use graph theory approaches to compare properties of functional networks built with EEG and link the observed behavioral data to those networks

## Materials and Methods

### Participants

Twenty-eight healthy adults (12 women, 16 men) aged 19 to 46 yr (mean 27.6 yr) participated in this study. All participants were informed about the task to be completed and provided written consent. The protocols in this study were approved by the Virginia Tech Institutional Review Board. All participants were tested for color blindness and corrected-to-normal vision. In addition, participants self-evaluated their hearing using guidelines from the American Speech-Language-Hearing Association. None of the participants reported any history of neurological disorders or hearing problems.

### Auditory Stimulus

**Fig 1:**
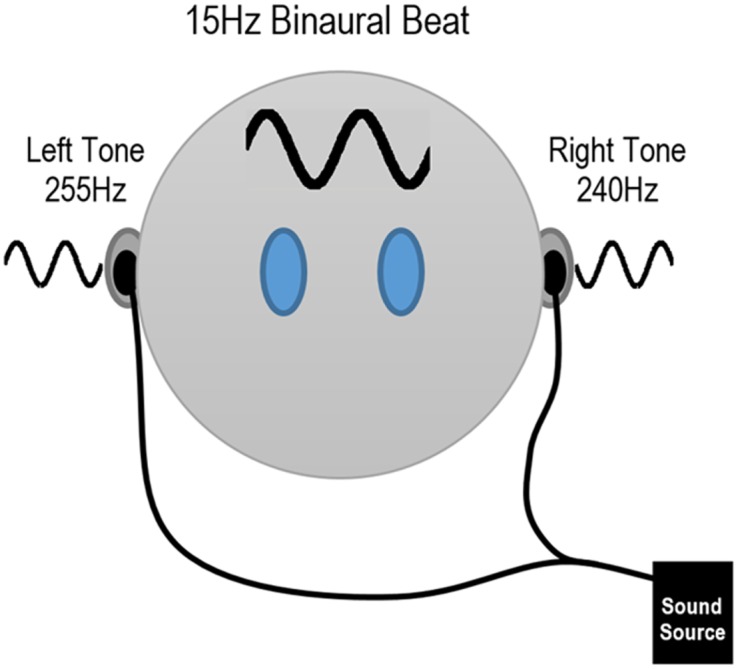
Example of a 15Hz Binaural Beat.

A battery of acoustic stimulation conditions were tested during the task. The three control conditions were 1) No Sound, 2) Pure Tone (R: 240Hz, L: 240Hz), and 3) Classical Music (Vivaldi—Spring). The three experimental conditions were 1) 5Hz Binaural Beat (R: 240Hz, L: 245Hz), 2) 10Hz Binaural Beat (R: 240Hz, L: 250Hz), and 3) 15Hz Binaural Beat (R: 240Hz, L: 255Hz). R and L indicate the frequency of the tones in the right and left ear respectively. The experimental binaural beats, 5Hz, 10Hz, and 15Hz, were chosen to represent the theta, alpha, and beta bands, respectively. Fig 1 shows an example of the 15Hz binaural beat. The tones were created in Matlab and presented to the participants using stereo headphones (MDR-NC7, Sony). Before the start of the experiment the volume of the auditory stimuli were set by the participants.

### EEG Recordings

A 16 gold cup passive electrode EEG system (OpenBCI, Inc., New York, NY) interfaced with LabVIEW was used to record the data at a sampling frequency of 128Hz. The locations of the electrode channels were Fp1, Fp2, F7, F8, F3, F4, T3, T4, C3, C4, P3, P4, O1, O2, Fz, Cz, which were placed using the 10-20 system [60]. The reference and ground electrodes were placed on the ear lobes. Electrodes were prepared with Ten20 EEG conductive paste (Weaver and Co., Aurora, CO) and electrode impedances were verified <5 k*Ω* prior to data collection. The testing took place in a quiet, dimly lit room.

### Visuospatial Task

**Fig 2:**
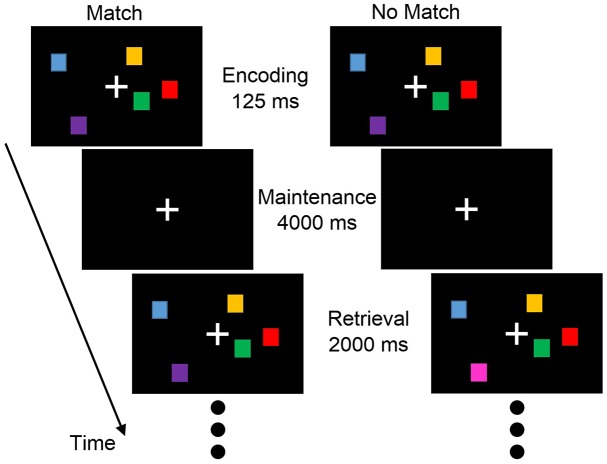
Delayed match-to-sample visuospatial working memory task.

The working memory task selected for this experiment was the delayed match-to-sample visuospatial task [8]. Fig 2 shows a match and no match trial. After encoding an initial image, the subject was instructed to retain the image in the absence of continuing input during working memory maintenance. During the retrieval process, the subject was asked to compare the retained and current image and to indicate whether they matched. Capacity, the limit on the ‘load’ that can be actively maintained, was calculated as

$$\begin{array}{c} K_{C}=C(H-F), \end{array}$$ (1)

where C is the load, H is the hit rate, and F is the false alarm rate. The hit rate is the percentage of correctly identified matches, and the false alarm rate is the percentage of non-matches identified as matches [8]. Thus, capacity measures how accurately the participants can identify correct matches scaled by the number of targets in each image.

The participants were seated comfortably in front of a computer monitor which presented the task. Two clearly marked buttons on the keyboard allowed the participant to indicate a match (left arrow) or a no match (right arrow) with their right hand. Before the start of the experiment, an initial load titration test was completed and involved an example practice round (one block of a 2-load task) and then proceeded to increasingly difficult loads (one block each of 3-, 4-, and 5-load versions of the task). The titrated load for the experimental task was determined by finding the load that produced the maximum capacity estimate for each individual participant. If two of the loads resulted in the same capacity, then the load with the highest hit rate was chosen.

After EEG preparations, the participant performed the experimental task at the load determined by the initial titration for 30 minutes. Every 5 minutes, the sound playing though the stereo headphones would change to one of the six different acoustic stimulation conditions. In order to minimize bias, all trials and all conditions were randomized over all participants. The task was presented using a custom script written with the Cogent Graphics Matlab toolbox.

### Behavioral Data Processing

The recorded behavioral data was processed for analysis using a custom Matlab script. First, trials on which the participant did not respond or pressed a non-target key were discarded (less than 5%). In order to effectively assess improvement due to oscillatory synchrony in the brain, it was important that each participant be tested at the limit of their individual working memory capacity. Therefore, the metrics used to compare behavior across acoustic stimulation conditions were Δ Accuracy and reaction time. Accuracy is defined as the number of correct trials both matches (Hit) and non-matches (Correct Rejection) divided by the total number of trials. Δ Accuracy is the difference between the accuracy at the end (3.5—5 mins) as compared to the beginning (0—1.5 mins) of the acoustic stimulation condition. Reaction time is the amount of time between when the target image appeared on the screen and when the participant hit a response button.

### EEG Data Processing

The raw EEG data was preprocessed using EEGlab [61]. First, the data were bandpassed filtered between 0.5Hz and 50Hz to remove drift and the 60Hz power line noise. Then, the filtered EEG was re-referenced to the average. Afterwards, the maintenance (125 ms—4125 ms, which corresponded to the time when no visuospatial array was present on the screen between the sample and probe stimuli) and retrieval (4125 ms—6125 ms) epochs were extracted. The retrieval epoch length remained constant even if the participant responded before the end of the 2 second interval. Finally, the baseline was removed (0-200 ms before stimulus presentation). Only correct trials (i.e. a Hit or Correct Rejection) were used. Epochs were inspected by hand for artifacts from eye blinks, movement, or other sources and were removed. The rejection rate was less than 5%.

Given that the goal was to analyze the overall brain configuration during the different acoustic stimulation conditions, regional links were determined by averaging the connections between the clusters of electrodes over the frontal, temporal, parietal, and occipital lobes. It should be noted that regions, in this context, refers to the average of the surface sites over the different cortices. All of the conditions were normalized against the No Sound results to show the changes in link strengths.

### Network Construction and Analysis

The processed EEG signals were filtered again, using EEGlab, into the four common frequency bands: theta (4Hz—8Hz), alpha (8Hz—12Hz), beta (12Hz—25Hz), and gamma (25Hz—40Hz) [62]. These filtered signals were then used to compute the time-frequency synchronization measure between channels. The graphical networks were constructed using the channels as the nodes and the time-frequency synchronization measure as the edge weights. Separate networks were created for the maintenance and retrieval epochs and the four different frequency bands.

#### Time-Frequency Synchronization Measure

A common measure of synchronization is called the Phase Locking Value (PLV). The PLV is a measure of the phase covariance between two signals. The PLV of two oscillators is 1 if the phase difference is continually fixed, and is 0 if constantly changing [63]. To compute the PLV, the EEG channel recordings were converted into analytic signals using a Hilbert transform from the Matlab Signal Processing Toolbox [64]. Then, the phase, in radians, of the *h*th channel in a given epoch, in either maintenance or retrieval, is denoted *ϕ*_*h*_(*t*). The phase difference between channel *h* and channel *i* is defined as

$$\begin{array}{c} \theta_{hi}\left(t\right)=(\phi_{h}\left(t\right)-\phi_{i}\left(t\right))\;\mathrm{mod}\;2\pi . \end{array}$$ (2)

Finally, to create the full graphical network, all pairs of channels are compared against each other via the Phase Locking Value

$$\begin{array}{c} {\text{PLV}}_{hi}=\frac{1}{N}[\sum_{k=0}^{N}\text{exp}(j\theta_{hi}\left(k\Delta t\right))], \end{array}$$ (3)

where $j=\sqrt{-1}$ is the imaginary unit, and $\Delta t=\frac{T}{N}$ where T is the epoch duration and N is total number of discrete steps.

#### Graphical Network Measures

Quantifying characteristics of the functional networks derived from neuroimaging data can be achieved using graphical network metrics [56, 65]. The graphical network is undirected and weighted and is constructed using the electrode channels as the nodes (*V* = {1, …, *n*}) and the PLV connection strength as the undirected edges (*E* = {(*i*, *j*) : ∃ an edge from *i* to *j*}). The network can be described using the adjacency matrix, *A*, and the edge weight matrix, *W*, defined as

$$\begin{array}{c} A=[\begin{array}{ccc} a_{11} & \ldots & a_{1n}\\ \vdots & \ddots & \vdots \\ a_{n1} & \ldots & a_{nn} \end{array}], \end{array}$$ (4)

$$\begin{array}{c} W=[\begin{array}{ccc} w_{11} & \ldots & w_{1n}\\ \vdots & \ddots & \vdots \\ w_{n1} & \ldots & w_{nn} \end{array}], \end{array}$$ (5)

where *n* = 16 and *a*_*ij*_ = 1 if (*i*, *j*) ∈ *E* and 0 otherwise. The elements of the symmetric weight matrix are $w_{ij}={PLV}_{ij}$ with the property that 0 ≤ *w*_*ij*_ = *w*_*ji*_ ≤ 1 for *i*, *j* = 1, …, *n*, *i* ≠ *j*.

The networks were analyzed using three common metrics: degree, clustering coefficient, and betweenness centrality. These metrics were computed using the Brain Connectivity Toolbox (BCT) in Matlab.

1. The degree of the *i*th node (D_*i*_) is the sum of the edge weights connected to the node. It is a measure of the amount of information coming into the node from other regions, and is computed using
  $$\begin{array}{c} D_{i}=\sum_{i=1}^{n}w_{ij}. \end{array}$$ (6)
2. The clustering coefficient of a node (${CC}_{i}$) is the proportion of the adjacent nodes which are interconnected. It can be considered a measure of local connectivity around the node and is defined by
  $$\begin{array}{c} {CC}_{i}=\frac{2}{k_{i}\left(k_{i}-1\right)}\sum_{j}^{n}\sum_{h}^{n}{\left({\tilde{w}}_{ij}{\tilde{w}}_{ih}{\tilde{w}}_{hj}\right)}^{1/3}, \end{array}$$ (7)
  where $k_{i}=\sum_{i=1}^{n}\left|\text{sgn}\left(w_{ij}\right)\right|$ is the unweighted degree. The weights are normalized, to ensure that the ${CC}_{i}$ remains between 0 and 1, using ${\tilde{w}}_{ij}=w_{ij}/\max_{i,j=1,\ldots ,n}w_{ij}$.
3. The betweenness centrality of a node (${BC}_{i}$) is the number of shortest paths between all nodes that pass through node *i*. A path of length *k* from node *j* to *h* is a sequence of *k* + 1 distinct nodes with consecutive nodes adjacent with respect to the edges in *E*. A shortest path between *j* and *h* minimizes *k*. The number of shortest paths from *j* to *h* is given by *σ*_*jh*_ and the number of those paths which include node *i* is given by *σ*_*jh*_(*i*). The betweenness centrality, defined as
  $$\begin{array}{c} {BC}_{i}=\sum_{j\neq h\neq i\in V}\frac{\sigma_{jh}\left(i\right)}{\sigma_{jh}}, \end{array}$$ (8)
  sums the fraction of shortest paths between nodes on which the *i*th node lies. High betweenness centrality indicates that the node has a large influence on overall transfer of information through the network.

#### Connectivity Ratio

In addition to the standard graphical network measures, a new metric, the connectivity ratio (CR), was defined to investigate the differences between the maintenance and retrieval networks. The 16 × 16 PLV matrices are averaged over all epochs for maintenance and retrieval separately, and the two average matrices which result are given by $PLV_{\text{Retrieval}}$ and $PLV_{\text{Maintenance}}$, respectively. To compute the CR, each the elements of the PLV retrieval matrix are is divided by the corresponding maintenance connection values elementwise and the resulting matrix is defined as CR. This metric provides a method of combining the two graphs into a single graph while maintaining valuable information about the continuity of the strength between them. Based on this definition, the lower the CR value the smaller the change in connection strengths between the maintenance and retrieval networks.

### Statistical Methods

The statistical software JMP was used to analyze both the behavioral and EEG data. Multiple ANOVAs were completed to analyze the behavioral response data and the network structures. Henceforward, CONDITION refers to the six acoustic stimulation conditions: None, Pure Tone, Classical Music, 5Hz BB, 10Hz BB, and 15Hz BB. BAND refers to the frequency band: theta, alpha, beta, and gamma. CHANNELS refers to the 16 individual channels of recorded EEG data. LINK refers to the Frontal—Temporal (F—T), Frontal—Parietal (F—P), Frontal—Occipital (F—O), Parietal—Occipital (P—O), Parietal—Temporal (P—T), and Temporal—Occipital (T—O) connections. The post hoc test chosen was the Tukey HSD, which was used to evaluate pairwise comparisons on the marginal means. The familywise error rate was kept at a maximum of 0.05.

For the behavioral data, the original dataset (*N* = 28) was bootstrapped 100 times. This number was chosen so it was on the same order of magnitude as the number of EEG data samples. An alpha level of 0.01 was chosen since all statistical analyses were completed on the bootstrapped behavioral dataset. In addition, the regional links were bootstrapped 100 times due to the large standard deviation of the dataset, and the alpha level was set to 0.0001, to be conservative.

Finally, ordinary least squares (OLS) regression was used to identify key metrics in understanding the relationship between the EEG and the behavioral data. The dependent variable was the Δ Accuracy. The independent variables included the maintenance and retrieval responses for degree, clustering coefficient, betweenness centrality, the regional link strengths, and the connectivity ratio. Each variable was bootstrapped 10,000 times so that the variation between the regressors and dependent variable could be accounted for. The regression was completed on the bootstrapped dataset. A correlation analysis determined that there is a high degree of multicollinearity between each of these metrics, as shown in S1 Fig. The correlation coefficient, r, for each pair is either strongly positive (yellow) or negative (blue). The high multicollinearity means that adding more than one parameter to the regression would be both redundant and insignificant, since all parameters would predict similar outputs. Therefore, multiple linear regression models and linear mixed models would not be appropriate for this analysis. Instead, each metric was evaluated separately, using OLS regression, to determine its ability to describe the recorded Δ Accuracy. The dataset, including the behavioral responses and the PLV connectivity networks, have been made publicly available [66].

## Results

### Working Memory Task Performance

**Fig 3:**
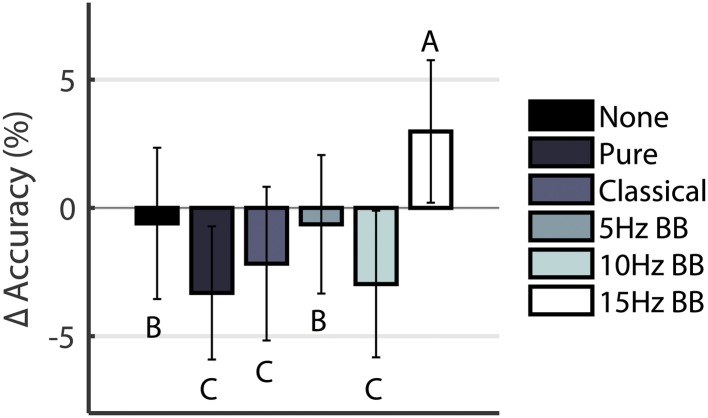
Δ Accuracy. Conditions marked with different letters are significantly different.

A one-way ANOVA showed that the effect of CONDITION on the Δ Accuracy was significant (F(5,594) = 67.184, p < 0.0001). Post hoc pairwise analyses are shown in Fig 3. Participants’ performance during the 15Hz BB was significantly more accurate over time than all other conditions. It was the only auditory stimulation condition that produced a positive change in accuracy over 5 minutes. All other acoustic stimulation conditions produce negative Δ Accuracy. However, no significant change occurred in the participants’ reaction time when compared in an ANOVA using CONDITION (F(5,594) = 0.194, p = 0.965).

### Connectivity Networks

**Fig 4:**
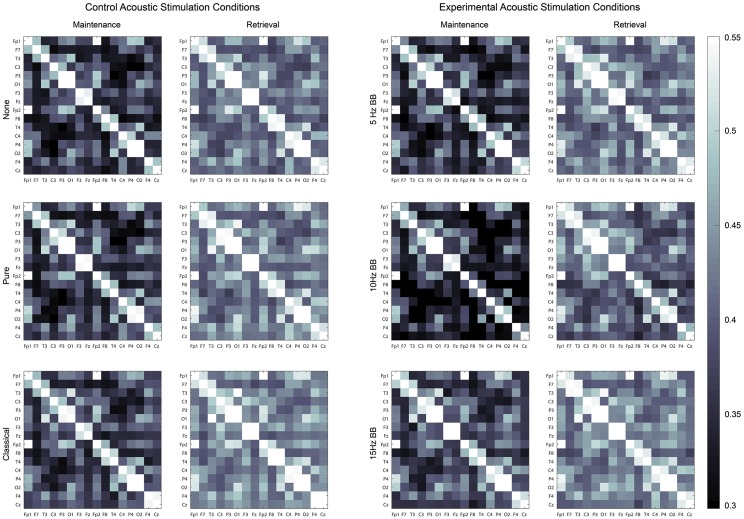
Theta band PLV connectivity network weight matrices. For each weight matrix, the diagonal is always equal to one.

The first one-way ANOVA examining the EEG data determined that the edge weights of the networks were significantly different between the maintenance (M = 0.472, SD = 0.037) and retrieval networks (M = 0.524, SD = 0.035) (F(1,2878) = 1180.243, p < 0.0001). Therefore, two separate 6 × 4 factorial ANOVAs, for maintenance and retrieval, were completed to determine the effect of CONDITION and BAND on the network structure. Both ANOVAs produced similar significant main effects, as shown in Table 1. For both maintenance and retrieval, the main effects of CONDITION and BAND were significant, but their interaction was not significant. Post hoc analyses revealed that the theta band had the largest activations in both the maintenance and retrieval segments (p < 0.0001). No significant effects were found in the other frequency bands. Henceforth, only the theta band will be examined for the rest of this analysis. Fig 4 shows the average PLV networks for the six conditions during both maintenance and retrieval as built by the EEG signal in the theta band.

**Table 1:** Results from the factorial ANOVA comparing Condition and Frequency Band.

	Maintenance		Retrieval	
	F-value	p-value	F-value	p-value
CONDITION	F(5,5736) = 53.1	p < 0.0001	F(5,5736) = 53.9	p < 0.0001
BAND	F(3,5736) = 199.1	p < 0.0001	F(3,5736) = 733.1	p < 0.0001
CONDITION × BAND	F(15,5736) = 0.9	p = 0.497	F(15,5736) = 1.1	p = 0.266

#### Connectivity Ratio

**Fig 5:**
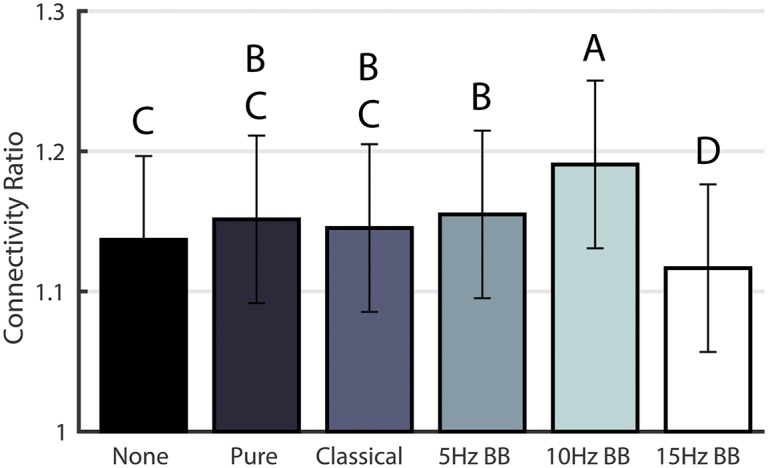
Connectivity Ratio for the six acoustic stimulation conditions. Conditions marked with different letters are significantly different.

The CR, computed from the weight matrices in Fig 4, were analyzed for a significant effect due to CONDITION using a one-way ANOVA. Based on the results, the acoustic stimulation type had a significant effect on the CR (F(5,1434) = 39.938, p < 0.0001). Significant post hoc pairwise analyses are represented by differing letters in Fig 5. The CRs resulting from the 10Hz BB and 15Hz BB conditions were significantly higher and lower, respectively, than all other conditions. This indicates that the change in connection strengths between the maintenance and retrieval networks was smallest for the 15Hz BB condition and largest for the 10Hz BB condition.

#### Graphical Network Measures

**Fig 6:**
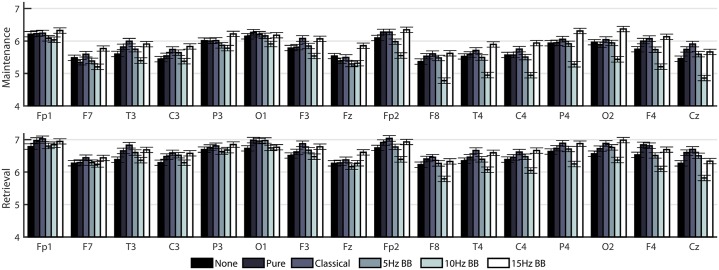
Mean degree of EEG nodes in cortical networks for all six acoustic stimulation conditions. Bars indicate standard error.

**Fig 7:**
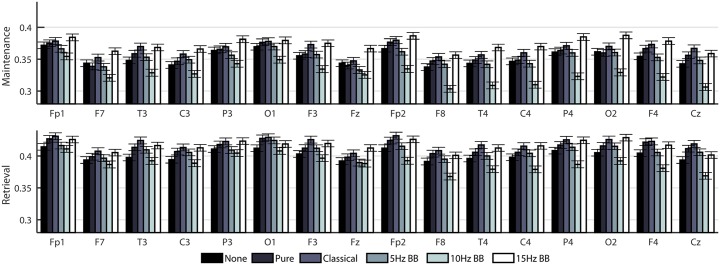
Mean clustering coefficient of EEG nodes in cortical networks for all six acoustic stimulation conditions. Bars indicate standard error.

**Fig 8:**
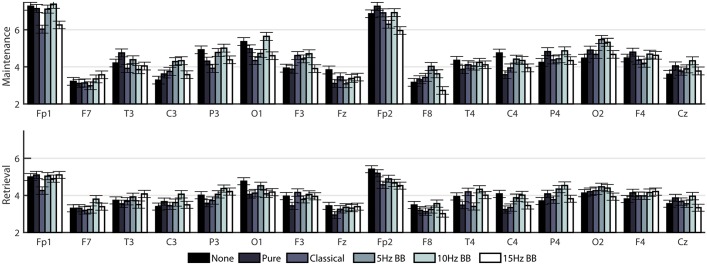
Mean betweenness centrality of EEG nodes in cortical networks for all six acoustic stimulation conditions. Bars indicate standard error.

To gain a more in-depth evaluation of the network structure in the theta band, two separate two-way ANOVAs (on the maintenance and retrieval segments) were constructed to compare the effect of CONDITION and CHANNELS on degree (Fig 6), clustering coefficient (Fig 7), and betweenness centrality (Fig 8). Table 2 shows the F-values from the ANOVAs. All p-values were less than 0.0001. For all network measures, the values from the two hemispheres are generally symmetric.

**Table 2:** Results from the 2-way ANOVA comparing Condition and Channels.

	Maintenance		Retrieval	
Metric	Channel	Condition	Channel	Condition
D	*F*(15,69536) = 57.7	*F*(5,69536) = 120.5	*F*(15,69536) = 34.9	*F*(5,69536) = 58.7
CC	*F*(15,69536) = 23.7	*F*(5,69536) = 147.1	*F*(15,69536) = 15.1	*F*(5,69536) = 73.1
BC	*F*(15,69536) = 143.8	*F*(5,69536) = 11.5	*F*(15,69536) = 37.5	*F*(5,69536) = 5.7

**Fig 9:**
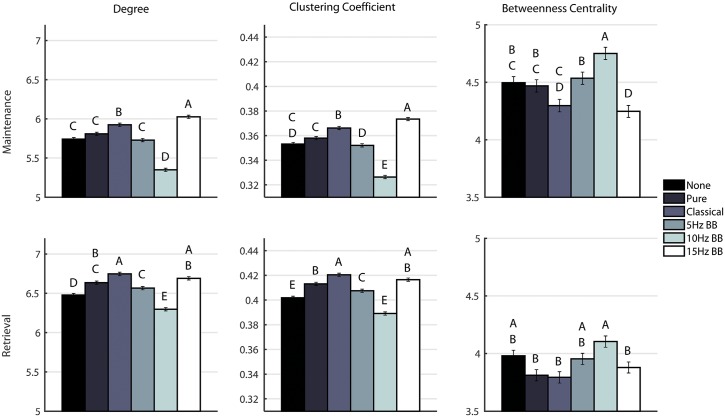
Comparison of degree, clustering coefficient, and betweeness centrality for each condition. Conditions marked with different letters are significantly different. The bars show standard error.

Fig 9 shows a comparison of the three metrics averaged over all the channels for each time segment. The significances were determined from the two-way ANOVAs described previously. For degree and clustering coefficient, the network measure is generally lower and higher for the 10Hz BB and 15Hz BB conditions, respectively, compared to all other conditions. Conversely, the betweenness centrality is higher and lower for the 10Hz and 15Hz BB conditions, respectively, compared to all other conditions.

#### Regional Link Strength

**Fig 10:**
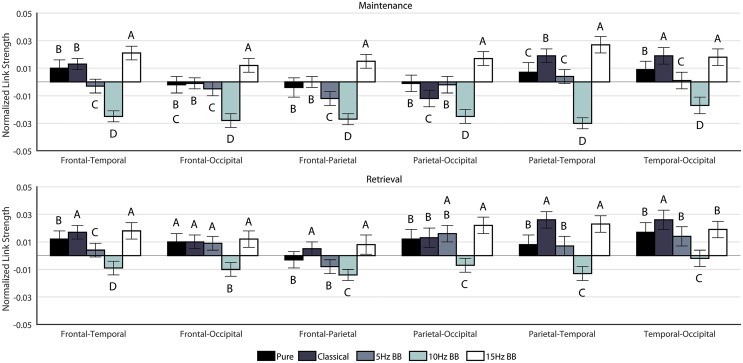
Regional Link Strengths. Each condition is normalized against None. The significances are shown for each link individually. Conditions marked with different letters are significantly different.

A 6 × 6 factorial ANOVA was constructed to compare the effect of LINK and CONDITION on the PLV link strengths for both maintenance and retrieval segments. Fig 10 shows the link strengths for both maintenance and retrieval. For both the maintenance and retrieval networks, the main effects of CONDITION, LINK and the interaction between CONDITION × LINK were significant, as shown in Table 3. Notably, the 15Hz BB connection strength values were significantly higher than all other conditions in all connections except for the Temporal—Occipital link during maintenance. This indicates that the 15Hz BB stimulus increased connectivity between the frontal lobe and all other brain regions as well as between the parietal lobe and all other brain regions. The connectivity pattern during retrieval were less clear, although the 15Hz BB link strength values were one of the highest conditions. Thus the effect of 15Hz BB on communication between brain regions is higher, and more distinguishable from other auditory stimuli, during maintenance than it is during retrieval of visuospatial stimuli.

**Table 3:** Results from the factorial ANOVA comparing Condition and Regional Link Strength.

	Maintenance		Retrieval	
	F-value	p-value	F-value	p-value
CONDITION	*F*(5,1764) = 2.09E + 3	p < 0.0001	*F*(5,1764) = 1.05E + 3	p < 0.0001
LINK	*F*(5,1764) = 1.17E + 4	p < 0.0001	*F*(5,1764) = 8.04E + 3	p < 0.0001
CONDITION × LINK	F(25,1764) = 48.2	p < 0.0001	F(25,1764) = 35.2	p < 0.0001

### Correlations Between Δ Accuracy and Network Topology

Table 4 displays, in order of predictive ability, the coefficient of determination (*R*^2^), intercept, and slope for each metric from the individual OLS regressions. All the metrics, except for betweeness centrality and CR, have a positive correlation with the behavioral changes. Based on the magnitude of the *R*^2^ values, the metrics computed during maintenance generally describe the change in behavioral changes better than those during retrieval.

**Table 4:** OLS Regression. M: Maintenance, R: Retrieval.

Metric	*R*^2^	Intercept	Slope
CR	0.230	0.836	−0.737
(P − O)_M_	0.210	−0.519	1.099
(F − P)_M_	0.185	−0.430	1.062
(F − O)_M_	0.171	−0.445	1.097
(P − T)_M_	0.145	−0.261	0.689
D_M_	0.134	−0.338	0.057
CC_M_	0.134	−0.301	0.817
BC_M_	0.128	0.305	−0.071
(F − T)_M_	0.122	−0.302	0.768
(F − P)_R_	0.088	−0.527	1.164
(T − O)_M_	0.081	−0.304	0.709
(P − O)_R_	0.079	−0.442	0.858
(P − T)_R_	0.076	−0.288	0.653
(F − T)_R_	0.051	−0.325	0.720
D_R_	0.047	−0.311	0.046
CC_R_	0.045	−0.274	0.644
(F − O)_R_	0.037	−0.340	0.741
(T − O)_R_	0.008	−0.130	0.258
BC_R_	0.006	0.077	−0.022

## Discussion

### 15Hz binaural beats increases accuracy in a visuospatial working memory task

Listening to 15Hz BB positively influenced the participants’ accuracy during the course of the 5 minutes by 3%. During all other conditions, the participants’ accuracy decreased by 1%—3%. No Sound and 5Hz BB produced a smaller decrease in accuracy while the Pure Tone, Classical Music, and 10Hz BB produced the largest decreases. This increase in performance of the working memory task can be explained by noting that 15Hz BB produces high synchronization within the auditory cortex [42] and falls within the beta band which is often associated with active concentration.

### Acoustic stimulation significantly affects the relative network connections during maintenance and retrieval in a visuospatial working memory task

Based on the results in Fig 5, 15Hz BB induces the smallest relative change in network connection strengths between the maintenance and retrieval portions of the working memory trials. Therefore, the networks are better preserved throughout the working memory task. Working memory maintenance is thought to be driven by reverberatory loops that allow sustained neuronal firing and thereby allow cognitive representations to be held in consciousness [67]. Consequently, sustained neural activity in appropriate networks is the hallmark of working memory task success, as seen in Table 4. The CR negatively correlates with the change in accuracy during the working memory task and has the highest *R*^2^ value of 0.23. Therefore, as the accuracy of the performance increases, the relative differences in the network activation between maintenance and retrieval decrease. To our knowledge, this paper is the first to demonstrate that 15Hz BB improves the consistency of relative connection strengths better than the other acoustic stimulation conditions and to use the CR to predict working memory task performance.

### Acoustic stimulation consistently impacts regional linkages during both maintenance and retrieval

The strengths of the regional connections offer some insight into the overall functional connectivity of the brain during the working memory task. In previous studies, the interactions between the parietal and prefrontal cortices have been strongly associated with working memory performance [10, 11]. As shown in Table 4, the frontoparietal connection for maintenance has a higher correlation (*R*^2^ = 0.185) with the performance than during retrieval (*R*^2^ = 0.088). Given the increase in working memory performance during exposure to 15Hz BB, we might infer that frontoparietal connectivity is more important during visuospatial working memory maintenance than during retrieval. This inference is consistent with the finding that parietal cortex is involved in storage of visuospatial information [68, 69] whereas the prefrontal cortex itself is important for executive control processes such as decisions made during retrieval. In addition, the regional links between P—O, F—O, and P—T for maintenance are on the same order of magnitude of the *R*^2^ value with the F—P link, as shown in Table 4. All of the links have a positive correlation with the behavior. Therefore, if the link strength increases, then working memory performance is positively affected as well.

### Stimulation condition significantly changes network structures by introducing edges that emphasize or de-emphasize the role of certain nodes

As shown in Figs 6–8, there is a high level of symmetry between the left and right hemispheres for the degree, clustering coefficient, and betweenness centrality. When the participants were surveyed after the completion of the session, the majority responded that they remembered the names of the colors which could account for the left hemisphere activation, which is associated with verbal processing. In addition, the channels with the highest values are Fp1, Fp2, F3, F4, P3, P4, O1, and O2 which correspond to the frontal, parietal, and occipital lobes. The 15Hz BB produces a high cumulative transfer of information over the whole network and edge weights which are homogeneously distributed. This is evidenced by Fig 9, which shows that 15Hz BB has a high degree and clustering coefficient in addition to a low betweenness centrality value. The low betweenness centrality indicates that all nodes are of more equal importance in the graph. Conversely, when the degree and clustering coefficient are low and the betweenness centrality is high, such as 10Hz BB, then the edge weights are not equally distributed and a few certain nodes are favored in the network. These data demonstrate that binaural beats significantly changes how edge weights, and therefore the structures in the network itself, are assigned. These metrics from the EEG data provide insight into the mechanism driving the behavioral findings that 15Hz BB improved working memory performance whereas 10Hz BB reduced working memory performance. It seems that a visuospatial working memory task is well served by increased communication across brain regions, particularly frontoparietal regions, and by consistency across nodes rather than increased strength in individual nodes.

In conclusion, listening to 15Hz binaural beats during a visuospatial working memory task can not only increase the response accuracy but also change the properties of the the cortical networks supporting task performance. A 3% increase in Δ Accuracy, over the 5 minutes, was found in participants who listened to the 15Hz binaural beat. All other acoustic stimulation conditions produced a negative change. In addition, the best predictor of the working memory performance is the connectivity ratio (CR), which indicates the relative change in network connection strengths between the maintenance and retrieval segments. During 15Hz binaural beats, the network characteristics are better preserved from the maintenance to the retrieval portions of each trial than the other acoustic stimulation conditions. This similarity in the network likely reflects the participants’ continued maintenance of the visuospatial pattern through the retrieval phase, when they must report the pattern held in mind. Finally, the 15Hz binaural beats produced the network with the most efficient data transmission. Therefore, a 15Hz binaural beat can be used to successfully augment working memory performance.

## Supporting Information

S1 Fig The correlation analysis between metrics used in the OLS regression. Matrix elements are pairwise correlation coefficients between metrics.
(EPS)
